# Electronic directly observed therapy (eDOT) therapy: a digital breakthrough in tuberculosis treatment – A commentary

**DOI:** 10.1097/MS9.0000000000000668

**Published:** 2023-04-18

**Authors:** Usha Kumari, Umaima Wasim, Sanjana Kumari, Freshta Khoshbakht

**Affiliations:** a Dow University of Health Sciences; b Ziauddin University, Karachi; c Herat Regional Hospital, Khaja Ali Movafaq Rd, Herat, Afghanistan

*Dear Editor*,

Tuberculosis (TB) is a challenging disease. It is endemic in low-income countries, where it is still a hot topic due to ineffective disease management, a lack of quality resources, and preventive efforts. TB is almost invariably curable with proper treatment. The most significant barrier to optimum TB therapy with current medications is adherence to the months-long drug regimen.

A key component of the anti-TB monitoring strategy typically endorsed by the WHO is directly observed treatment (DOT), which encourages medical staff, community volunteers, or family members to witness and record the patient taking each dosage^1^. It remains an irrefutable fact that DOT has significantly reduced the global effect of TB. But it has some major pitfalls preventing its universal implementation. DOT is expensive and labor-intensive. Therefore, it requires immediate attention for its use, especially in low- and middle-income countries^2^. According to a study conducted by Queiroz^3^, patients reported having to break their isolation due to the need to go to the primary care unit to take medication. Furthermore, there are inconsistencies in the incentives provided, requirements to miss work, and communication issues between patients and healthcare. Patients are dissatisfied with healthcare professionals and feel ashamed, humiliated, and helpless^3^. This highlights the fact that TB is a serious public health issue that necessitates an effective approach.

Electronic Directly Observed Therapy (eDOT), also known as Virtual Directly Observed Therapy DOT is a newly emerging approach to treat TB. It works in such a way that a member of a medical team can watch a patient swallow an anti-TB drug, using real-time video or a prerecorded video that is submitted for later confirmation. Guo^4^ found that videophone observation is a reliable method of observing medication administration with improved adherence and without TB recurrence. Furthermore, the utility of eDOT has become prominent during the COVID-19 pandemic. However, there is no difference in adherence to eDOT in the pre-Covid and post-Covid eras^5^.

eDOT is a patient-centric strategy to assist patients. It is a time- and cost-effective method of treatment^4^ and it allows the patient to take the drug freely at the desired time and place^6^. eDOT is a resource-efficient way to provide direct observation for anti-TB treatment and minimizes the burden on TB-endemic countries^6^,^7^. Healthcare providers think mobile health technology can improve patient care because of its ease of access^8^.

The digital world is rapidly evolving. With the increasing proportion of the population that possesses devices such as mobile phones, computers, or tablets with video capabilities, the likelihood of trained observers using electronic devices to activate eDOT is also increasing. There is currently a scarcity of universally applicable, adaptable, and effective strategies for monitoring and supporting treatment compliance. eDOT is still an emerging technology and it needs to be evaluated under more versatile conditions to determine its capability. Healthcare authorities and stakeholders should take measures to increase TB awareness and improve access to TB treatment.

## Ethical approval

NA.

## Consent

NA.

## Source of funding

None to declare.

## Conflicts of interest disclosure

The authors report no actual or potential conflicts of interest.

## Data availability statement

No data is associated with this submission.

## Provenance and peer review

Not commissioned, externally peer reviewed.
